# Andrographolide ameliorates d-galactosamine/lipopolysaccharide-induced acute liver injury by activating Nrf2 signaling pathway

**DOI:** 10.18632/oncotarget.17149

**Published:** 2017-04-17

**Authors:** Chen-wei Pan, Shou-xing Yang, Zhen-zhen Pan, Bo Zheng, Jian-zhang Wang, Guang-rong Lu, Zhan-xiong Xue, Chang-long Xu

**Affiliations:** ^1^ Department of Infectious Disease, The Second Affiliated Hospital and Yuying Children's Hospital of Wenzhou Medical University, Wenzhou, Zhejiang, 325027, China; ^2^ Department of Gastroenterology, The Second Affiliated Hospital and Yuying Children's Hospital of Wenzhou Medical University, Wenzhou, Zhejiang, 325027, China; ^3^ Department of Infectious Disease, The First Affiliated Hospital of Wenzhou Medical University, Wenzhou, Zhejiang, 325027, China

**Keywords:** andrographolide, LPS, D-GalN, Nrf2, NF-κB

## Abstract

Andrographolide (ADH), a diterpenoid lactone extracted from *Andrographis paniculata*, has been found to have anti-inflammatory and anti-oxidative effects. However, its protective effects and mechanisms on liver injury have not been investigated clearly. This study takes an attempt to reveal the protective effects and mechanism of ADH on lipopolysaccharide (LPS) and D-galactosamine (D-GalN)-induced acute liver injury in mice. The mice liver injury model was induced by LPS (60 mg/kg) and D-GalN (800 mg/kg), and ADH was given 1 h after LPS and D-GalN treatment. Hepatic tissue histology was measured by H&E staining. Serum alanine aminotransferase (ALT) and aspartate aminotransferase (AST) levels were detected by detection kits. The levels of TNF-α and IL-1β were detected by ELISA. Moreover, malondialdehyde (MDA) and reactive oxygen species (ROS) contents were also detected. Meanwhile, the expression of Nrf2, HO-1, and NF-κB were detected by western blot analysis. The results showed that ADH treatment improved liver histology and decreased the levels of ALT, AST, MPO, IL-1β, TNF-α, as well as MDA and ROS levels of hepatic tissues in a dose-dependent manner. ADH also inhibited LPS/D-GalN-induced NF-κB activation. The expression of Nrf2 and HO-1 were increased by treatment of ADH. In conclusion, ADH protected against LPS/D-GalN-induced liver injury by inhibiting NF-κB and activating Nrf2 signaling pathway.

## INTRODUCTION

Acute liver injury is an inflammatory condition that characterized by the sudden loss of hepatic function [1]. It has a high rate of mortality [1, 2]. LPS has been identified as a major factor that leads to liver injury [3]. Meanwhile, D-GalN has the ability to aggravate LPS-induced liver injury in only in a few hours [4]. Furthermore, LPS and D-GalN-induced mice liver injury model are widely used as experimental animal models to investigate human liver injury [5]. In this model, LPS significantly up-regulates the expression of inflammatory cytokines, such as TNF-α and IL-1β [6]. These cytokines can cause serious liver tissue injury such as acute liver injury [7]. Till now, few methods are applied to cure acute liver injury except for liver transplantation [8]. Therefore, developing a high-efficient drug for acute liver injury is pressingly needed. Nrf2 is an important transcription factor that up-regulates cytoprotective genes in response to oxidative stress [9]. Previous studies showed that Nrf2 was involved in the development of liver injury [10]. Nrf2 has been identified as a potential target for the treatment of liver injury.

Andrographolide (ADH), an active constitutent isolated from *Andrographis paniculata*, has been found to have anti-inflammatory and antioxidant effects [11]. ADH has been reported to inhibit LPS-induced acute lung injury in mice [12]. ADH also prevented inflammatory bone loss *in vivo* [13]. *In vitro*, ADH has been reported to inhibit LPS-induced inflammatory mediator production in RAW264.7 cells [14]. ADH also inhibited HMGB1-induced inflammatory responses in human umbilical vein endothelial cells [15]. Previous studies showed that ADH exhibited its anti-inflammatory effects by inhibiting NF-κB activation [16]. However, the protective effects and mechanisms of ADH on LPS and GalN-induced acute liver injury remain unclear. Therefore, this study takes an attempt to investigate the effects of ADH on liver injury and reveal its mechanism.

## RESULTS

### ADH ameliorates LPS/GalN-induced liver histopathologic changes

**Figure 1:**
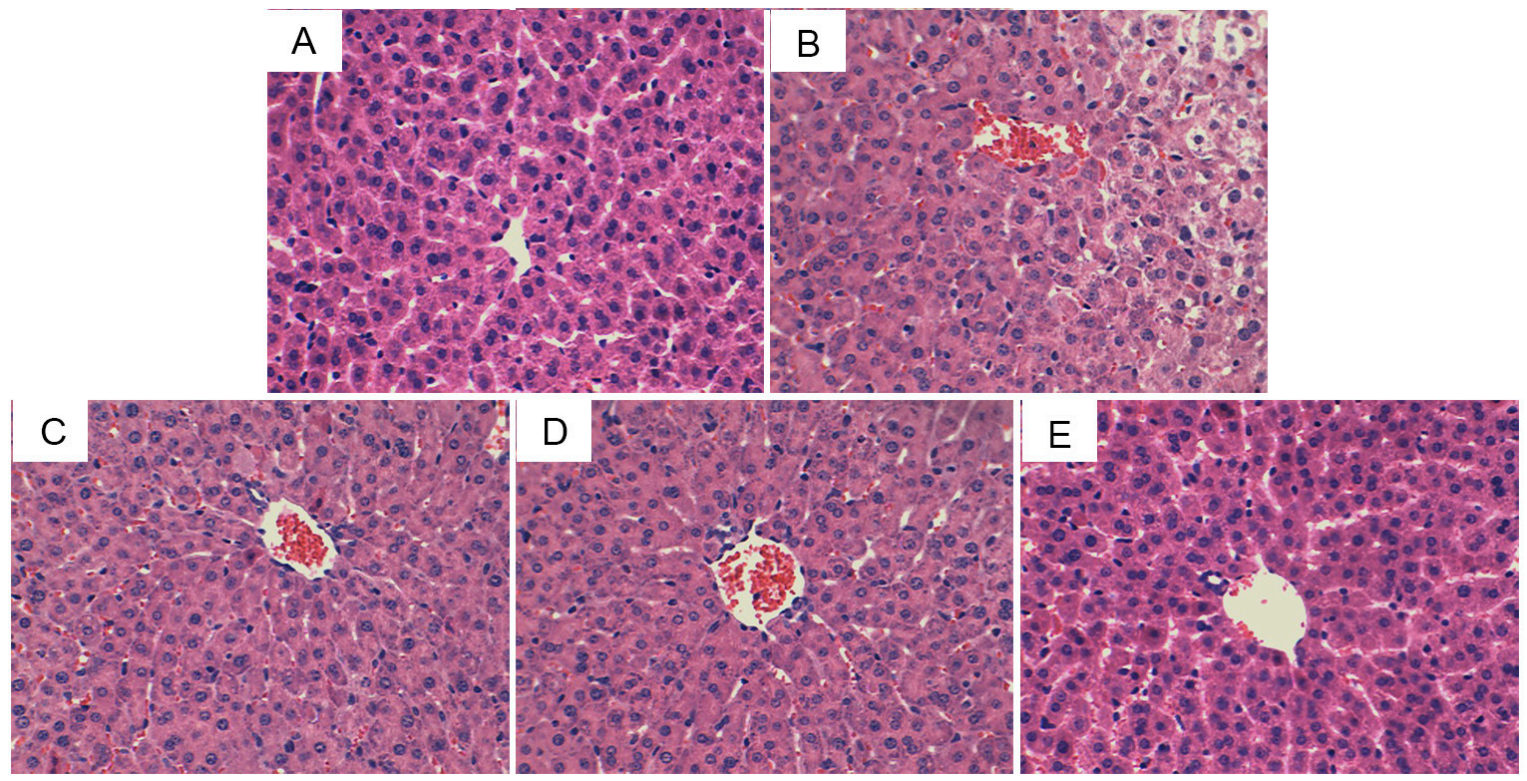
Effects of ADH on histopathological changes in liver tissues Representative histological changes of liver obtained from mice of different groups. **(A)** Control group, **(B)** LPS/GalN group, **(C)** LPS/GalN + ADH (2.5 mg/kg) group, **(D)** LPS/GalN + ADH (5 mg/kg) group, **(E)** LPS/GalN + ADH (10 mg/kg) group (Hematoxylin and eosin staining, magnification 200×).

The effects of ADH on LPS/GalN-induced liver histopathologic changes were measured by H&E staining. The results showed that liver tissues of the control group exhibited an integral hepatic lobular architecture and normal hepatocytes (Figure 1A). However, liver tissues of LPS/D-GalN group showed disturbed architecture, including extensive hemorrhage, necrosis and neutrophil infiltration (Figure 1B). These histopathologic changes induced by LPS/D-GalN were significantly inhibited by ADH (Figure 1C, 1D, 1E).

### ADH attenuates LPS/GalN-induced serum ALT and AST levels

**Figure 2:**
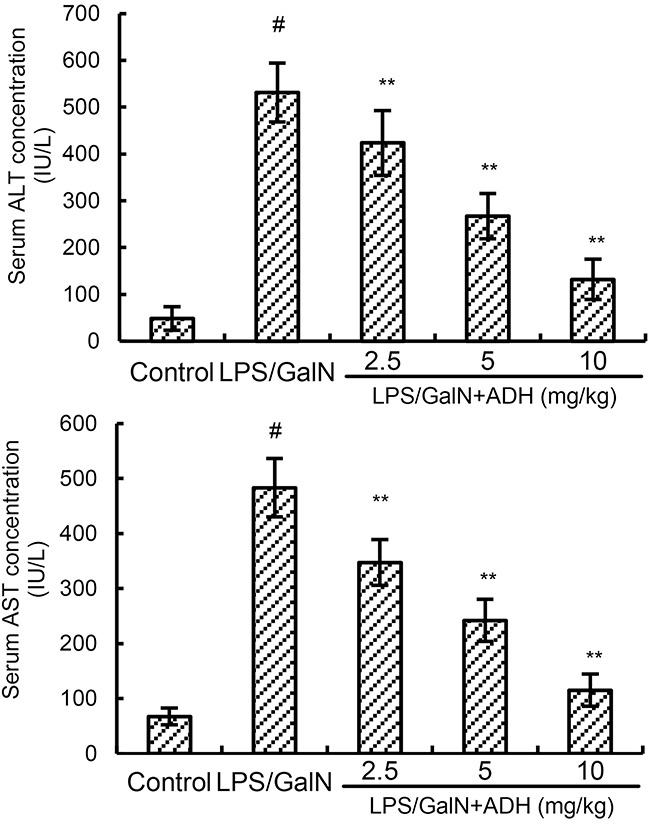
Effects of ADH on serum ALT and AST levels ADH (2.5, 5, and 10 mg/kg) were administered intraperitoneally 1 h after LPS/GalN treatment. 8 h after LPS/GalN challenge, the levels of ALT and AST were detected. The values presented are the mean ± S.E.M. of three independent experiments. P^#^<0.01 vs. control group, P*<0.05, P**<0.01 vs. LPS/GalN group.

The effects of ADH on LPS/GalN-induced serum ALT and AST levels were measured in this study. As shown in Figure 2, there was a marked increase in the production of serum ALT and AST in LPS/GalN group. However, the increases of ALT and AST production induced by LPS/GalN were significantly inhibited by ADH (Figure 2).

### ADH attenuates LPS/GalN-induced MPO, MDA and ROS levels

**Figure 3:**
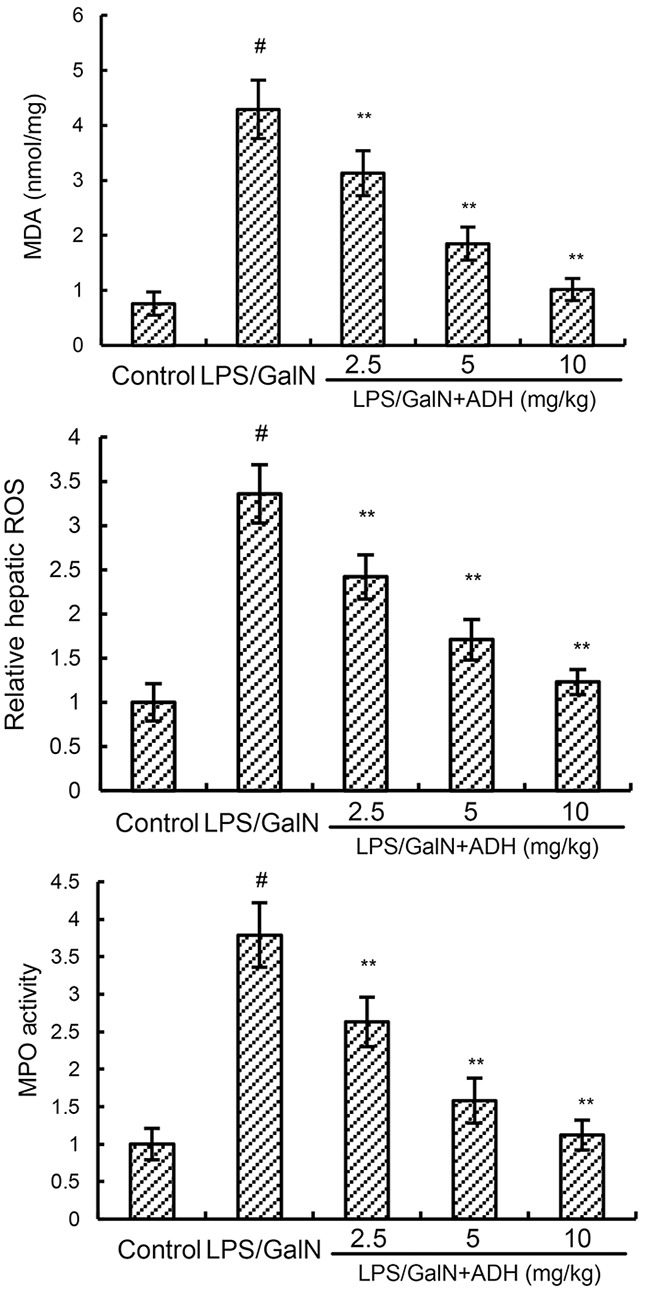
Effects of ADH on liver MPO, MDA, and ROS levels ADH (2.5, 5, and 10 mg/kg) were administered intraperitoneally 1 h after LPS/GalN treatment. 8 h after LPS/GalN challenge, the levels of MDA and ROS were detected. The values presented are the mean ± S.E.M. of three independent experiments. P^#^<0.01 vs. control group, P*<0.05, P**<0.01 vs. LPS/GalN group.

To investigate the anti-oxidative effects of ADH, the effects of ADH on ROS and MDA production were detected in this study. As shown in Figure 3, there was a significant increase in the production of MDA and ROS in LPS/GalN group. However, the increases of MDA and ROS production induced by LPS/GalN were significantly inhibited by ADH (Figure 3). The effects of ADH on MPO activity were detected in this study. As shown in Figure 3, there was a significant increase in the production of MPO in LPS/GalN group. However, the increase of MPO activity induced by LPS/GalN were significantly inhibited by ADH (Figure 3).

### ADH inhibits LPS/GalN-induced hepatic TNF-α and IL-1β production

**Figure 4:**
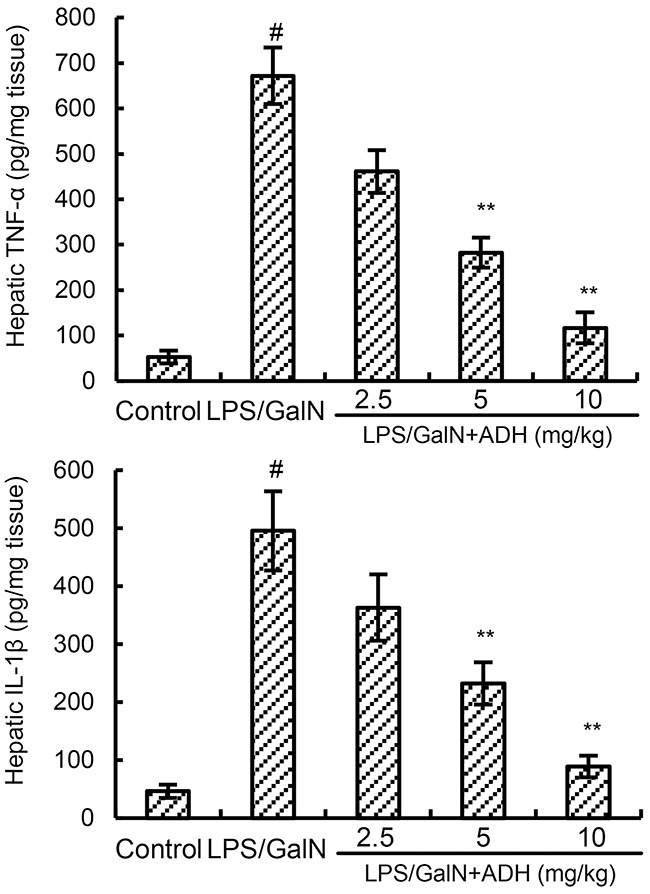
Effects of ADH on hepatic TNF-α and IL-1β levels ADH (2.5, 5, and 10 mg/kg) were administered intraperitoneally 1 h after LPS/GalN treatment. 8 h after LPS/GalN challenge, the levels of hepatic TNF-α and IL-1β were detected. The values presented are the means ±S.E.M. of three independent experiments. P^#^<0.01 vs. control group, P*<0.05, P**<0.01 vs. LPS/GalN group.

To investigate the anti-inflammatory effects of ADH, the effects of ADH on inflammatory cytokines TNF-α and IL-1β production were detected in this study. As shown in Figure 4, there was a marked increase in the production of TNF-α and IL-1β in LPS/GalN group. However, the increases of TNF-α and IL-1β production induced by LPS/GalN were significantly inhibited by ADH (Figure 4).

### ADH inhibits LPS/GalN-induced NF-κB activation

**Figure 5:**
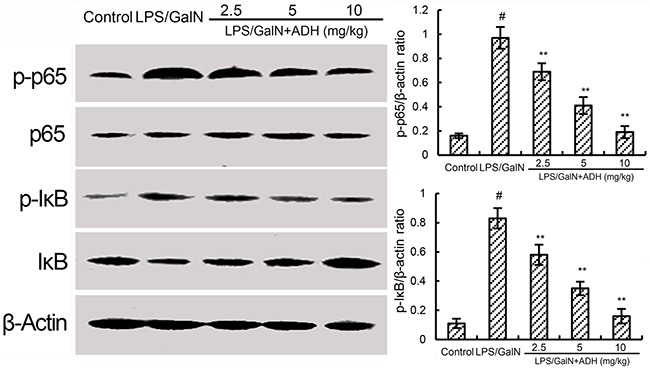
Effects of ADH on LPS/GalN-induced NF-κB activation ADH (2.5, 5, and 10 mg/kg) were administered intraperitoneally 1 h after LPS/GalN treatment. 8 h after LPS/GalN challenge, NF-κB activation were detected. The values presented are the mean ±S.E.M. of three independent experiments. The density values of the Western blot were normalized for β-actin. P^#^<0.01 vs. control group, P*<0.05, P**<0.01 vs. LPS/GalN group.

NF-κB, a critical transcription factor, has been known to play an important role in the regulation of inflammatory cytokines. To investigate the anti-inflammatory mechanism of ADH, the effects of ADH on LPS/GalN-induced NF-κB activation were detected in this study. The results showed that LPS/GalN significantly up-regulated the levels of phosphorylation of NF-κB and IκBα. However, treatment of ADH dose-dependently inhibited LPS/GalN-induced NF-κB activation (Figure 5).

### Effects of ADH on Nrf2 and HO-1 expression

**Figure 6:**
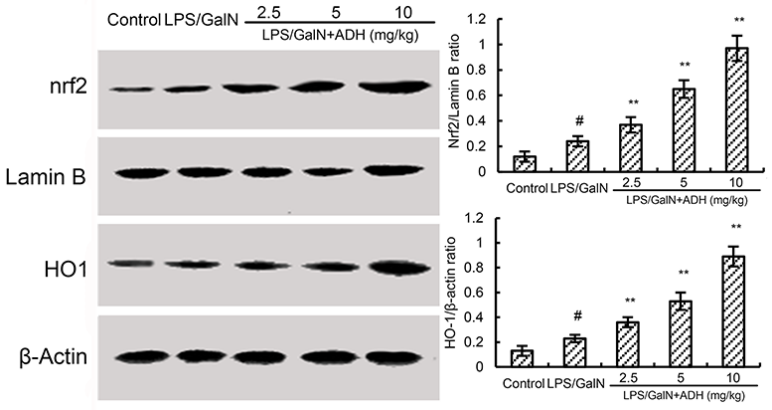
Effects of ADH on Nrf2 and HO-1 expression ADH (2.5, 5, and 10 mg/kg) were administered intraperitoneally 1 h after LPS/GalN treatment. 8 h after LPS/GalN challenge, Nrf2 and HO-1 expression were detected. The values presented are the mean ±S.E.M. of three independent experiments. The density values of the Western blot were normalized for β-actin. P^#^<0.01 vs. control group, P*<0.05, P**<0.01 vs. LPS/GalN group.

Nrf2 is a transcription factor implicated in the transactivation of gene coding detoxifying enzymes. To investigate the anti-oxidative mechanism of ADH, the effects of ADH on Nrf2 signaling pathway were measured in this study. As shown Figure 6, the expression of Nrf2 and HO-1 were up-regulated by treatment of LPS/GalN. However, compared to the LPS/GalN group, the expression of Nrf2 and HO-1 in LPS/GalN + ADH groups increased significantly.

## DISCUSSION

Previous studies showed that ADH has anti-inflammatory and anti-oxidative effects. In this study, we investigated the protective effects of ADH on LPS/GalN-induced liver injury. And the results of our present study showed that ADH inhibited LPS/GalN-induced liver injury by inhibiting NF-κB and activating Nrf2 signaling pathway. ADH might be considered as a potential therapeutic reagent for the treatment of liver injury.

LPS/GalN-induced liver injury is characterized by severe inflammatory responses. Elevated inflammatory cytokine productions were observed in mice of LPS/GalN-induced liver injury [17]. These inflammatory cytokines, especially TNF-α and IL-1β, have been known to play critical roles in the pathogenesis of liver injury [18, 19]. Studies showed that inhibition of these inflammatory cytokines could attenuate LPS/GalN-induced liver injury in mice [20]. In this study, we found that ADH significantly inhibited LPS/GalN-induced TNF-α and IL-1β production. NF-κB is an important transcription factor that regulates inflammatory cytokine expression during immune and inflammatory responses [21, 22]. Once activation, NF-κB p65 translocates from cytoplasm into the nucleus to regulate inflammatory cytokine expression [23]. Previous studies showed that NF-κB activation is associated with pathological liver injury-induced by LPS/GalN [24]. Furthermore, many herbal medicines protected against LPS/GalN-induced liver injury by inhibiting NF-κB activation [25, 26]. In this study, we detected whether the anti-inflammatory mechanism of ADH was through inhibiting NF-κB signaling pathway. The results of the present study showed that ADH inhibited LPS/GalN-induced liver injury by inhibiting NF-κB activation. The results suggest that ADH inhibited LPS/GalN-induced inflammatory cytokine production by inhibiting NF-κB activation.

Oxidative stress also plays an important role in the development of LPS/GalN-induced liver injury [27]. LPS/GalN-induced liver injury is characterized by elevated ROS and MDA levels in liver tissues [28]. In this study, our results showed that ADH significantly inhibited LPS/GalN-induced ROS and MDA production in liver tissues. These results indicated that ADH could inhibit LPS/GalN-induced oxidative stress in mice. Nrf2 has been reported to play critical roles in the regulation of oxidative response [29, 30]. Previous studies showed that Nrf2 was involved in the development of liver injury [31]. Nrf2-/- mice showed more severe pathological changes in the liver when the mice were treatment of arsenic [32]. In addition, activating of Nrf2 signaling pathway could inhibit liver injury produced by various hepatotoxicants. Many herbal medicines protected against LPS/GalN-induced liver injury by activating Nrf2 signaling pathway [33]. Nrf2 has been identified as a potential target for the treatment of liver injury [34]. In this study, our results showed that treatment of ADH up-regulated the expression of Nrf2 and HO-1. These results suggested that ADH protected LPS/GalN-induced liver injury by activating Nrf2 signaling pathway.

In conclusion, our results suggested that ADH had protective effects against LPS/GalN-induced liver injury in mice. The protective effects may be related to the inhibition of inflammatory and oxidative response. ADH inhibited LPS/GalN-induced liver injury by inhibiting NF-κB and activating Nrf2 signaling pathway.

## MATERIALS AND METHODS

### Reagents

ADH (purity>98%) was purchased from the National Institute for the Control of Pharmaceutical and Biological Products (Beijing, China). LPS and GalN were obtained from the Sigma Chemical Co. (L-2880, St. Louis, MO, USA). The kits for biochemical analysis of ALT, AST, and MDA were purchased from the Jiancheng Bioengineering Institute of Nanjing (Nanjing, China). IL-1β and TNF-α ELISA kits were purchased from Biolegend (CA, USA). Antibodies against TLR4, p65, p-p65, p-IκBα, and IκBα were purchased from Santa Cruz Biotechnology (Santa Cruz, CA). The second antibody was provided by Cell Signaling Technology Inc. (Beverly, MA, USA). All other chemicals were of reagent grade.

### Animals

BALB/c mice (6–8 weeks old, weighing approximately 18-22g) were purchased from the Center of Experimental Animals of Wenzhou Medical University (Wenzhou, China). The mice were housed in microisolator cages under a 12/12 h light/dark cycle at 24±1 °C and 40–80% humidity. The mice were fed with food and water *ad libitum*. All animal experiments were performed in accordance with NIH guidelines for the care and use of laboratory animals.

### Experimental protocol

Sixty mice were divided into five experimental groups (n=10) as follows: Control group; LPS/GalN group, LPS/GalN + ADH (2.5, 5, and 10 mg/kg) groups. Mice were injected intraperitoneally with LPS (60 mg/kg) and D-GalN (800 mg/kg) to establish the mice liver injury model [35]. ADH (2.5, 5, and 10 mg/kg) were administered intraperitoneally 1 h after LPS/GalN treatment. The blood and liver tissues were collected 8 h after LPS/GalN challenge for subsequent analysis. The chose of 8 h after LPS/GalN challenge were based on previous study [36].

### Histological examination

The liver tissues were collected 8 h after LPS/GalN treatment. Then, the tissue samples were fixed in 10% buffered formalin for 24 h. The samples were dehydrated with graded alcohol, embedded in paraffin, and sectioned. Finally, the samples were stained with hematoxylin and eosin according to standard procedures.

### Biochemical assays

Blood samples were collected 8 h after the LPS/GalN challenge. The levels of ALT and AST in serum were detected using the detection kits (Jiancheng Bioengineering Institute of Nanjing) according to the manufacturer's instructions. Relative hepatic ROS level was detected as previously described [37]. The content of liver MPO and MDA were detected by using test kits (Jiancheng Bioengineering Institute of Nanjing) according to the manufacturer's protocols.

### ELISA assay

The liver tissues were collected 8 h after LPS/GalN treatment. Then the tissues were weighed and homogenized with PBS (1:9, w/v). The levels of inflammatory cytokines TNF-α and IL-1β in the supernatants were detected by ELISA (Biolegend, USA) according to the manufacturer's instructions.

### Western blot analysis

The liver tissues were collected 8 h after LPS/GalN treatment. Total proteins from liver tissues were extracted with T-PER protein extract kit (Pierce, Rockford, IL, USA) according to the manufacturer's instructions. The concentration of the protein was determined by BCA protein assay kit (Beyotime, China). Equal amounts of protein were separated through 12% SDS polyacrylamide gels. Subsequently, the proteins were transferred to PVDF membranes. The membranes were blocked with 5% non-fat milk for 2 h at room temperature. Then, the membranes were incubated with primary antibodies: TLR4, p65, p-p65, p-IκBα, and IκBα. After washing three times with TBST, the membranes were probed with HRP-conjugated secondary antibody and visualized by the ECL western blotting detection system.

### Statistical analysis

All experimental data were expressed as mean ± SEM. Multiple comparisons were evaluated by one-way ANOVA the Tukey-Kramer method were used. Statistical significance was defined as P<0.05.
